# Race and Patient-Physician Communication on Blood Pressure Management: Helping Improve Care

**DOI:** 10.1007/s11886-025-02294-9

**Published:** 2026-01-27

**Authors:** Samar A. Nasser, Ashley Pender, Ayan Ali, Kardie Tobb, Keith C. Ferdinand

**Affiliations:** 1 Department of Clinical Research & Leadership, School of Medicine and Health Sciences, The George Washington University, Washington, DC USA; 2 Department of Medicine, Jefferson Heart Institute, Sidney Kimmel Medical College, Thomas Jefferson University Hospital, Philadelphia, PA USA; 3 Department of Medicine, University of Pennsylvania Perelman School of Medicine, Philadelphia, PA USA; 4 Cone Health, Greensboro, NC USA; 5 Department of Medicine, Tulane University School of Medicine, New Orleans, LA USA; 6 Section of Cardiology John W. Deming Department of Medicine, Tulane University School of Medicine, 1430 Tulane Ave, 70112 New Orleans, LA USA

**Keywords:** Race, Communication, Hypertension, Black patients, Disparities, Social determinants of health

## Abstract

**Purpose of Review:**

To provide an overview of the relationship of race and ethnicity and patient-physician/clinician communication on blood pressure management.

**Recent Findings:**

The suboptimal interactions experienced by patients from underrepresented racial and ethnic populations may be improved by enriching relationships among clinicians, population health scientists, policymakers, and community members/leaders. Current care communication and policies for managing hypertension appear to be insufficient. Implementing and disseminating effective strategies to efficiently improve the quality of hypertension care and recognize social determinants of health among minoritized populations is intertwined with enhanced communication.

**Summary:**

Approaches such as self-affirmation—encouraging patients to reflect on their strengths and values—can enhance patient-clinician communication. The use of standardized blood pressure management protocols and technology-based interventions (such as telehealth or mobile health apps) can improve healthcare communication and outcomes. Integration of cultural humility and sensitivity training, and engagement with community-based organizations positively impact patient-physician/clinician communication and resultant blood pressure reduction and control. Future research should focus upon implementation of policies to prioritize solutions to overcome communication barriers, with an emphasis on equity and the unique needs of minoritized populations.

## Introduction

Cardiovascular disease (CVD) is the leading cause of death worldwide, posing a major public health concern with significant impact on morbidity and mortality [1]. Of the potential CVD risk factors, hypertension is the most potent and prevalent risk factor [2]. Estimated United States (U.S.) health care costs for hypertension in 2016 were $79 billion (95% CI, $72.6–$86.8 billion), and approximately half of American adults have elevated blood pressure (BP) and nearly 50% of these patients are uncontrolled [2]. There are potentially many effective strategies for controlling hypertension. Nevertheless, in the U.S. control is approximately 25% and even less so in certain racial and ethnic populations, and especially among non-Hispanic Black (NHB) males [3]. Although race and ethnicity are social terms, with no scientific validity, suboptimal BP control, especially in NHB patients, may reflect suboptimal patient-physician/clinician communication. Effective communication fosters trust, self-efficacy, and better self-management, all of which are linked to improved medication adherence and BP control [4]. When patients feel heard and understood, they are more likely to follow treatment plans and engage in healthy behaviors, underscoring the importance of the shared decision-making style [4, 5].

Minoritized populations may experience a higher prevalence, greater disease severity, and overall worse hypertensive outcomes [2, 3, 5]. Despite longstanding awareness of inequities and attempted initiatives to address them, disparities have persisted for decades [6]. Improving equity in CVD care and delivery must address the complex and multifactorial causes, including socioeconomic factors, patient-physician/clinician relation factors, healthcare system inadequacies, and to a much lesser extent, biological considerations [1, 6]. A comprehensive approach across multiple stakeholders, including systemic, organizational, community, policy, clinician, and patient engagement is needed. By exploring shortcomings and proposing targeted solutions, this review highlights potentially effective approaches for equitable and individualized hypertension management. Analysis of the intersection of race, communication, and hypertension control and the specific challenges faced by NHB patients are addressed. Finally, solutions are proposed to overcome barriers to improve communication in care for effective and equitable hypertension management across racial and ethnic populations.

## Race and Hypertension in the United States

Hypertension is the leading preventable CVD risk factor for global mortality, causing over 10 million deaths annually [2]. In the U.S., NHB individuals experience approximately four times the hypertension-related CVD mortality of White individuals [6]. In addition to NHB individuals exhibiting higher hypertension rates, they also endure early CVD onset, and increased CVD mortality compared to White individuals [7]. Hypertension, defined as systolic blood pressure (SBP) of 130 mm Hg or greater or a diastolic blood pressure (DBP) of 80 mm Hg or greater, is present in an estimated 120 million adults in the U.S. (48.1%) and the majority, 92.9 million (77.4%) are uncontrolled [8, 9]. The southeastern region of the U.S. has the highest prevalence of hypertension [8]. When assessing the national prevalence by racial and ethnic subgroups, NHB adults have the highest rates of hypertension (56%), followed by non-Hispanic White adults (48%), non-Hispanic Asian adults (46%), and Hispanic adults (39%) [9]. In the U.S., approximately 1 in 5 people with high BP have kidney disease, and Black Americans are almost 4 times more likely to have kidney disease than White Americans [10, 11]. Black patients have earlier hypertension onset, greater severity, and ultimately, worse CVD outcomes [10].

Although race is a sociocultural construct, it entails a toxic fusion of intergenerational and individual exposures, lived experiences, sociocultural influences, and historical policies and inequities in resources that affect disadvantaged groups creating adverse health outcomes. The National Academies of Sciences, Engineering, and Medicine (NASEM) has assessed challenges in the use of race and ethnicity in genomics research [12] and researchers are urged to exclude race as a proxy for describing human genetic variation [13]. Nevertheless, in population health research, U.S. life expectancy differs drastically by location, economic situation, and race and ethnicity [14]. According to the “*Ten Americas”* analysis, there is continued existence of remarkably different Americas within the U.S. in life expectancy, dramatically depending on social determinants of health (SDoH) and one’s racial and ethnic identity. These persistent health disparities were exacerbated by the COVID-19 pandemic and serve as a critical indicator of a population’s health. The life expectancy gap across the ten Americas increased from 12.6 years in 2000 to 20.4 years in 2021 [14]. Consequently, race remains beneficial as an identifier in population research to expose and address health disparities and injustices that Black and other minoritized people experience. This evidence reinforces the need to address SDoH as a clinical priority, reshaping care strategies to bridge these deadly divides especially given that SDoH contribute to BP control. Not surprisingly, race-unaware risk prediction models calculate risk well for White individuals, but may less accurately predict risk for minoritized populations potentially underestimating the impact of race and racism on mortality and suboptimal care [15]. A concerted effort is needed to eliminate disparities by disseminating evidence-based equitable health care, education, and employment, and confronting factors that fuel inequalities.

## Social Determinants of Health

Understanding the factors that contribute to CVD disparities is essential to advancing health equity. SDoH influence a patient’s potential to realize their full health [16], and account for a significant portion of hypertension-related CVD burden. Historic discriminatory policies and structural racism have led to disparities in socioeconomic status, healthcare access, education, food security, housing stability, and environmental factors—all of which contribute to the prevalence of hypertension and differences in outcomes [16]. Exposure to systemic racism, discrimination, and clinician implicit bias can erode trust, reduce patient satisfaction, and negatively affect communication quality [17]. Poor-quality communication—such as less empathy, more clinician verbal dominance, and less patient-centeredness—has been directly linked to lower medication adherence and worse hypertension control, particularly among Black patients [17]. For patients facing severe structural barriers (e.g., lack of healthy food, unsafe environments), the influence of patient-clinician communication on hypertension outcomes may be diminished, as these broader SDOH can overwhelm the benefits of good communication. Overall, underrepresented racial and ethnic groups make up 40% of the total U.S. population, and the adverse effects of SDoH can severely undermine health outcomes [18]. Historical discrimination, perpetrated by prior longstanding, unjust policies and institutional practices, contributes to structural racism, further limiting equitable and innovative health care for NHB individuals. Moreover, CVD outcomes exist beyond the zip code or built environment. These include both measured and unmeasured variables: racism, clinician bias, adverse long standing cultural patterns (often sold to and promoted) such as marketing cigarettes in Black communities, sugar-based beverages, corner stores, fast-food establishments, and targeting the Southern diet [16]. Integration of the community’s voice can provide insight into protective factors that may influence health behaviors and outcomes. Communites are also influenced by social structures and attempts at recreating or challenging the social structures can shift the pace and flow of change in communities [16]. Ultimately, addressing SDOH is inseparable from improving patient-clinician communication in hypertension management. Community empowerment and partnerships trickle down to the patients, and can impact the structural barriers to enhance communication.

## Genetics & Hypertension

Although the SDoH account for most disparities in hypertension prevalence and control, there may be genetic and biological contributions to these differences. Accordingly, Black patients demonstrate higher levels of the gene encoding apolipoprotein L1 (APOL1), associated with focal segmental glomerulosclerosis and hypertensive nephrosclerosis [19]. The APOL1 gene with the risk alleles are also associated with higher SBP and earlier onset of hypertension in Black patients [19, 20]. In addition, elevated lipoprotein(a) (Lp[a]), an independent risk factor for CVD, is more prevalent in Black individuals and associated with increased arterial stiffness and hypertension [20, 21].

Genetic variations in the intrarenal renin-angiotensin-aldosterone system (RAAS) have also been observed among Black and White adults with various environmental exposures. In response to a high sodium intake, some Black adults may not suppress RAAS as effectively as White adults despite similar activation levels with low salt intake. Overall, these are potentially linked to genetic polymorphisms in the angiotensinogen gene, and the interplay of genetic variants may also potentiate their impact on hypertension, end-stage renal disease and cardiovascular events in Black patients [22]. Notwithstanding these considerations in genetic background, the suboptimal hypertension control in Black patients and worse outcomes are mainly driven by the SDoH impacting the mortality gap between Black and White individuals [18, 23]. Prior studies have attributed differences in BP by race to a combination of genetic, environmental, societal, and behavioral factors but have stopped short of estimating the relative contribution of each due to challenges in measuring the influence of structural racism and other social inequities. Rao and colleagues evaluated the association of genetic West African ancestry with BP response and cardiovascular outcomes among Black participants in the Systolic Blood Pressure Reduction Intervention Trial (SPRINT) [24]. SPRINT, a clinical trial that randomized participants to intensive (< 120 mm Hg) or standard (< 140 mm Hg) systolic BP control, presented a unique opportunity to study blood pressure response by genetic ancestry because all participants in the trial were afforded equal access to providers and treatments for the duration of the study. Among self-reported Black individuals enrolled in SPRINT, the trajectories of BP, kidney function, and left ventricular mass over time were not different across tertiles of the proportion of West African ancestry, however a higher proportion of West African ancestry was associated with a modestly lower risk for CV events. Thus, extrinsic and structural societal factors, more than genetic ancestry, may be the major drivers of racial disparity in cardiovascular health associated with hypertension [24].

## Barriers & Potential Solutions to Disparities in Hypertension Control

**Fig. 1:**
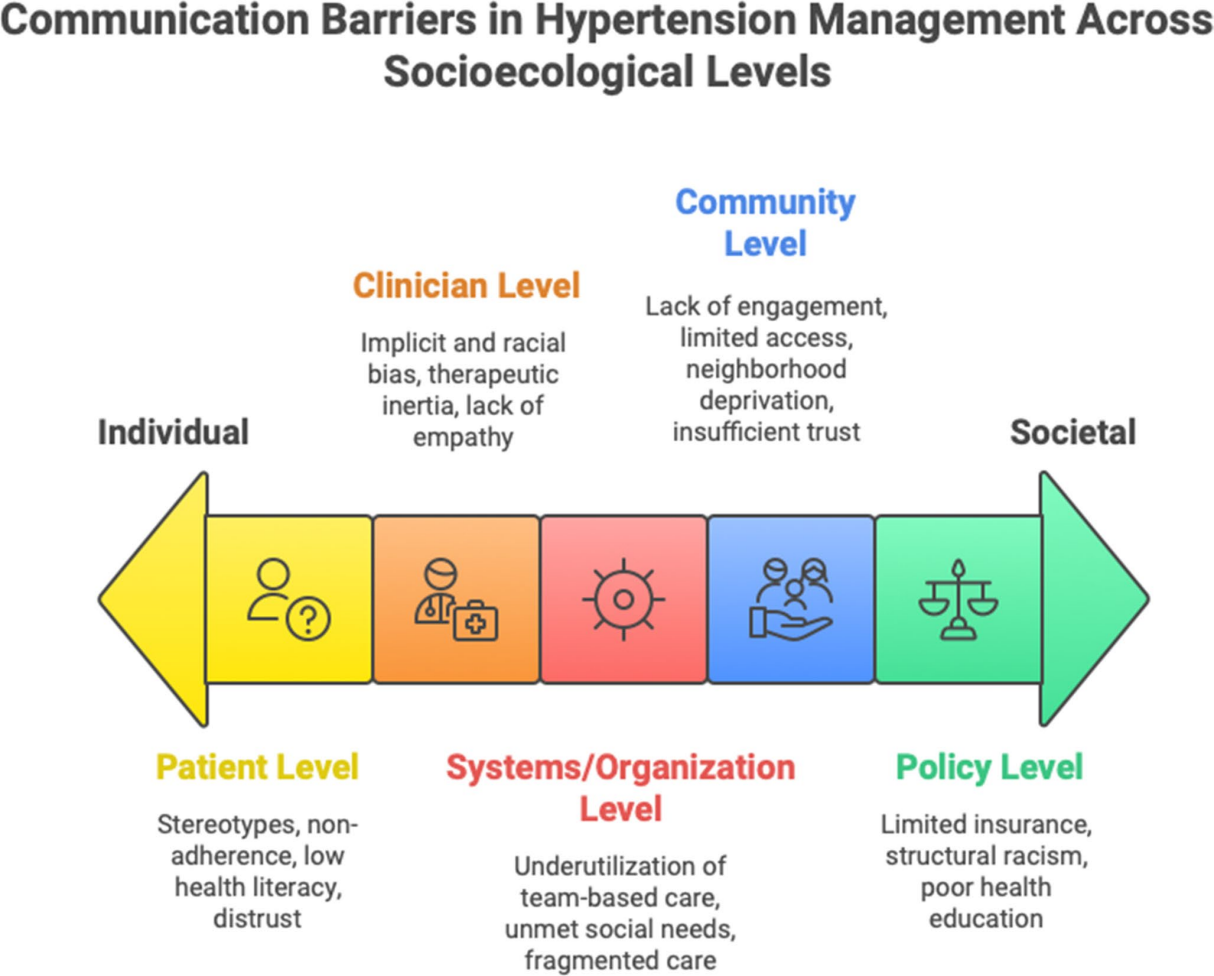
Barriers to communication challenges in hypertension management: barriers at multiple levels—patient, clinician, organizational, community, and policy—to promote equitable hypertension management. References: Figure created with data from [25, 44, 53, 72]

Disparities in hypertension control are shaped by a complex interplay of factors spanning from individual patient behaviors to broader policy frameworks. Herein, the socioecological framework was used as a guide to identify barriers to the successful implementation of BP control communication interventions [25] (Fig. 1). Effective communication strategies must address these barriers at multiple levels—patient, clinician, organizational, community, and policy—to promote equitable hypertension management. Multifaceted interventions, tailored to the needs of diverse populations and supported by robust communication strategies, are essential to achieving equitable blood pressure control and improving cardiovascular health outcomes. Lack of patient trust in the medical system is associated with worse health outcomes, stemming from racism, discrimination and unethical research practices [26]. Practicing cultural humility and developing shared-decision-making enhance clinician-patient communication and relationships and actively seeking to understand patient’s cultural beliefs which are needed to overcome distrust [10, 26, 27]. These methods may be incorporated by engaging community leaders to understand local health needs, respecting patient’s beliefs, providing accessible language interpretation, while concurrently addressing underlying clinician bias/stereotypes [26]. Addressing disparities in hypertension control demands a multifaceted, socioecological approach that recognizes and intervenes on communication barriers at the patient, clinician, organization, community, and policy levels to engage a team-based, interactive approach to patient care [27].

### Patient Level

Stereotypes can shape perceptions, attitudes, and behaviors toward others resulting in discrimination and health disparities. Stereotype threat refers to the internal fear (and threat of) being perceived negatively based on identity-related social stereotypes causing a psychological and physiological response that may influence health and healthcare experiences [28]. Minoritized patients are at greater risk of experiencing stereotype threat which has been linked to decreased memory and increased anxiety [29–31]. These associations undermine the therapeutic alliance central to patient-clinician communication and lead to underutilization of care and non-adherence [32–34]. In contrast, encouraging anxiety-coping strategies (pre-prepared questions and inviting family/support to visits) and incorporating affirmations in the clinical encounter demonstrates a commitment to open communication. Similarly, displaying anti-discriminatory systems/policies demonstrates a commitment to equity [35, 36]. Pharmacological and lifestyle non-adherence are significant barriers to hypertension control in minoritized populations. Low health literacy, complex regimens, absence of refill synchronization, and competing demands are factors that are central to communicating and engaging with patients [37]. An investigational pilot study in NHB patients with uncontrolled hypertension, Text NOLA, demonstrated that respect for patients’ backgrounds with a culturally-sensitive communicative approach via bidirectional electronic messaging could improve social support, medication adherence and BP control, without changes in pharmacotherapy [38]. To improve adherence, and in consideration of low health literacy, individualized and repeated clinician and pharmacist-led education interventions should be initiated at the time of diagnosis [39, 40]. Often, patients bear the burden of complex medication regimens. Consolidating medication regimens using combination pills/ polypills and limiting daily dosing are feasible ways to improve adherence [40, 41]. Also, refill synchronization, which is the ability of patients to pick up all of their medications in a single visit improves adherence [42]. Finally, multiple competing demands may be addressed with medication therapy communication reminders in the forms of phone calls, text messages, smart phone/device apps and self-monitoring blood pressure to cue patients to take medications [38, 39, 43].

Self-measured BP monitoring in patient-centered care models increases accuracy of blood pressure assessment and engages patients in their health management [44]. According to a secondary analysis of a randomized clinical trial, individualized patient self-monitoring of BP and self-titration of medication may be an effective way to improve BP control, as compared to usual care [45]. Thus, a potential solution to increase opportunities to attend health visits involves supplementing in-person visits with telemedicine and digital technologies to increase opportunities for patient engagement [45–48].

### Clinician Level

**Fig. 2:**
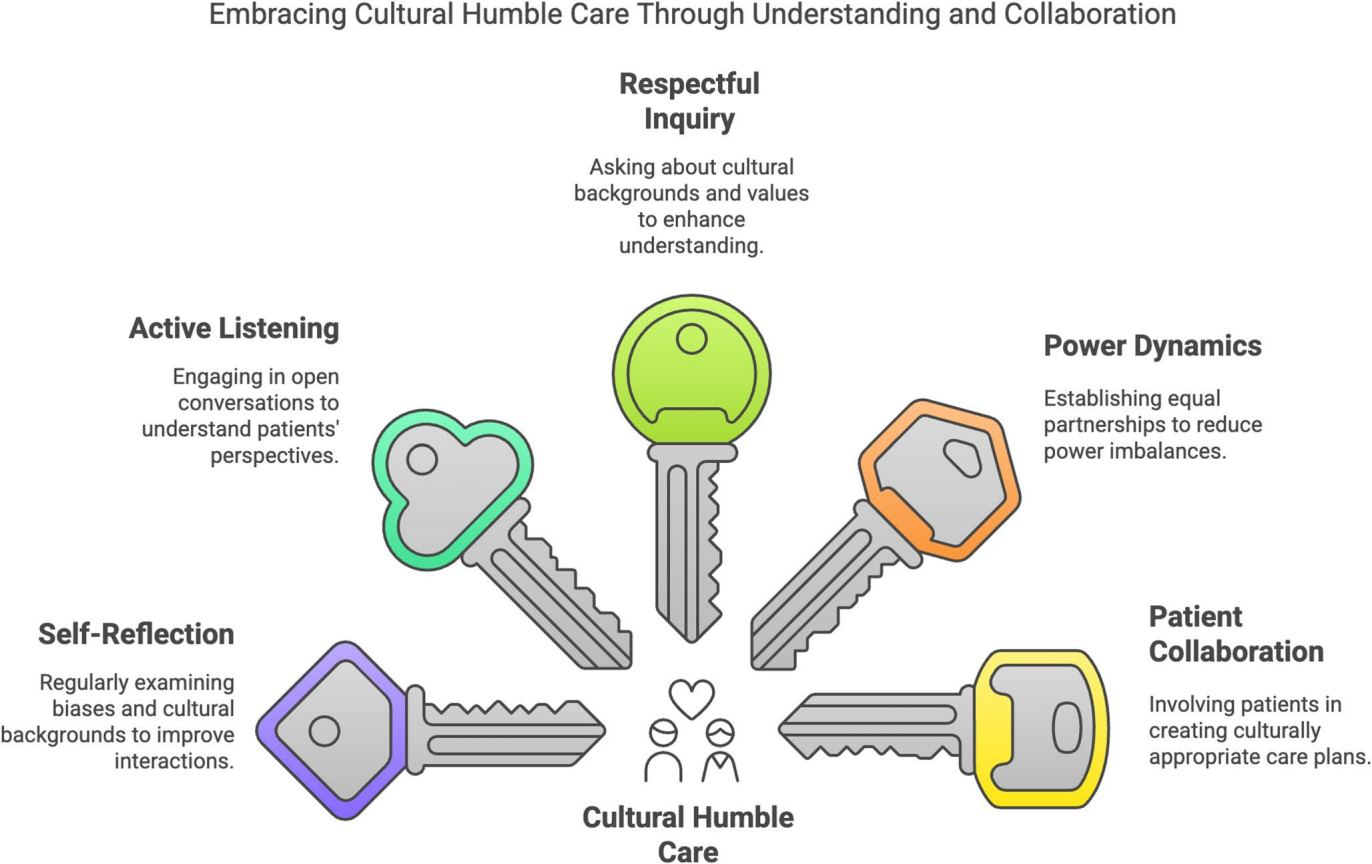
Strategies to implement culturally sensitive care. Implementing culturally competence and cultural humility allows clinicians to create a more inclusive, patient-centered approach that acknowledges the complexity of cultural identities and promotes better health outcomes for diverse populations. References: Figure created with data from [39, 53, 76]

Implicit and explicit racial bias and discrimination are pervasive and deeply rooted in U.S. society and contribute to health inequity. Racial bias informs clinical decision-making and is well documented in the literature [49–51]. Compared to White counterparts, Black patients receive lower quality care independent of disease status, insurance, and health care setting. There are racial differences in patient-clinician interactions with Black patients experiencing less empathy and less respect [52, 53]. These encounters with less humility undermine the therapeutic relationship and promote distrust and highlight the need for cultural humility. Ferdinand and colleagues characterize cultural humility as, “self-reflection and self-critique, learning from patients (avoiding cultural stereotyping), developing and maintaining respectful partnerships, and actively continuing these positive relationships” [53]. A critical method to decrease stereotype threat involves countering racial prejudice with cultural humility and implicit/explicit bias training of both clinical and non-clinical staff [50, 53]– [54]. Additionally, improving communication by providing clinicians and staff with training in verbal communication, critical feedback, and most importantly the delivery of clear verbal and written communication is necessary [54]. Patient’s perceptions are positively influenced by incorporating cues that minority groups are valued, including diverse staff, and displaying images of racial role models [35, 53]. Therefore, integrating culturally sensitive approaches by listening and ensuring that patients’ concerns are taken seriously, irrespective of their race are critical processes that offset racial bias (Fig. 2) [35, 55]. Additionally, implementing anti-bias training and raising awareness of implicit and explicit bias counters racial bias [49, 50].

One form of racial bias that occurs during clinical assessments relates to patient credibility. Notably, Black patients are less likely than others to be believed or to have their concerns taken seriously [50], a manifestation of testimonial injustice. This epistemic injustice involves the patient (i.e., speaker) who is unfairly judged on the credibility of his/her issue by the clinician (i.e., hearer), exposing a suppressed injustice in health care interactions. Accordingly, a study revealed that one of the most prominent experiences of disrespect for Black patients was being dismissed, disbelieved, or not taken seriously by their doctors [50]. To validate this finding, Lee and colleagues identified credibility-related language in the electronic health record (EHR) and found that clinicians used language undermining credibility substantially more often among Black compared with White patients [49]. The evidential terms can reflect doubt and were more commonly found in the Black patient records indicative of racial bias in clinician/physician assessment of patient credibility. Recent attention is focused upon eliminating stigmatizing EHR language, including language undermining patient credibility to unmask the roots of this language and confront the underlying problem of racial bias, including the tendency to dismiss or disbelieve Black patients. In general, incorporating cultural humility and cultural competence into training, emphasizing self-reflection and learning from patients fosters respectful partnerships which enhance clinician-patient relationships [53]. Furthermore, by standardizing EHR language to avoid undermining patient credibility, particularly for Black patients and training clinicians to recognize biased language in documentation leads to a greater appreciation of the issues surrounding racial bias and uprooting the underlying causes [49].

Therapeutic inertia and bias/discrimination are key clinician-level factors in hypertension management negatively affecting Black and other minoritized patients. Therapeutic inertia, or clinical inertia, is the lack of initiation or up-titration of therapy when treatment goals are unmet. In a 16,114-participant study conducted at safety-net hospitals in the San Francisco Health Network, race-based differences in therapeutic inertia were demonstrated. Black patients had the lowest treatment intensification and lowest hypertension control. Treatment intensification was associated with 21–26% of the racial differences in BP control [51]. A secondary analysis of the Systolic Blood Pressure Intervention Trial (SPRINT) involving 8,557 participants highlighted standardized BP management protocols to mitigate race-based therapeutic inertia and provide more equitable care. In the SPRINT Trial, therapeutic inertia prevalence was similar or lower for NHB participants as compared to non-Hispanic White participants [52]. Application of standardized antihypertensive medication treatment protocols with improved communication, education and quality care initiatives, and financial strategies for large-scale dissemination and implementation demonstrated equitable treatment intensification and optimized healthcare outcomes among Black patients [44, 51].

### Systems/Organization Level

Health systems bear responsibility for mitigating ongoing issues to help address unmet social needs and ensure that the gap in hypertension control is closed. Groups of interventions and activities need to be organized to help health systems develop deliberate strategies to enhance their hypertension management [56]. For instance, lack of collaboration among the healthcare team and EHR integration leads to fragmented care [57, 58]. Organizations can leverage teams consisting of patient navigators, pharmacists, nurses and physicians to enchance communication among teams and improve BP management. Lee and colleagues proposed a successful model for BP management that included a mechanism to identify patients, patient training in home BP monitoring, and patient self-blood pressure monitoring. The patient-collected data was then integrated into the EHR along with a BP treatment algorithm that facilitated patient navigator BP titration. Lastly, additional support was provided by pharmacists, nurse practitioners/medical doctors with the patient referred back to the primary care practitioner/referring physician once BP was optimized or for escalation of therapy for complex cases [58–60]. Potential solutions to underutilization of EHR/clinical decision support should integrate clinical decision support systems (CDSS) to promote equitable care by incorporating guidelines/best practice advisories into the EHR [61]. Moreover, incorporating prompts for medication titration when consecutive blood pressures are not at goal [62] and ensuring patient recorded measurements are integrated into the EHR) leads to less emphasis on individual decisions and more standardized systems of care across patient populations.

Failure to recognize and address various SDoH results in an unrelenting, longstanding mortality gap between Black and White individuals. These persistent CVD disparities and the associated reduction in life expectancy has led to an unacceptably U.S. high financial and social burden [63]. A potential solution to insufficient efforts to address SDoH involve partnering with community-based organizations (CBOs) and enabling shared goals between health systems and CBOs [57, 64]. Additionally, screening patients for intervenable SDoH and implementing solutions to recognize barriers can be feasibly implemented. For example, among patients who have limited access to transportation, supplementation with telemedicine and digital technologies in-between the in-person visits increase opportunities for patient engagement [46–48]. Relative to the lack of access to culturally competent/culturally-sensitive care, provider training on cultural humility and partnering with CBOs can ensure care is addressing the unique needs of NHB other minoritized communities [44, 64].

### Community Level

Community-based organizations must collaborate with healthcare systems to move beyond the existing transactional models and promote more relationship-oriented, culturally sensitive interactions. Recently, the CDC foundation led a project to develop an inclusive report *Recommendations for Strengthening Partnerships* between health departments and community-based organizations to address health inequities [64]. It is important for health systems and CBOs to align their goals and processes to address unmet social needs. Literature demonstrates that most health care organizations relied on CBOs to improve their patient’s social needs and health care organizations should tailor their relationships with CBOs based upon shared goals [65]. Developing the connection between local health departments and CBOs will help build trust, thereby paving the way to equitable clinical outcomes. Accordingly, Mills and colleagues revealed that a community-based intervention for engaging predominantly Black churches led to improved cardiovascular health and reduced racial inequities [66]. Although a lack of community engagement, and related insufficient trust between clinicians and Black communities, leads to poorer health outcomes, building relationships with community leaders (i.e., through churches or other trusted organizations) increases engagement and improves health outcomes [66]. Moreover, CBOs are uniquely positioned to improve social support for patients [67], and future consideration should focus upon community-based participatory research to explore social support interventions in hypertension management.

Furthermore, a lack of access to preventive and health-promoting services is a barrier to BP management [68]. Without access to preventive care and health promotion community programs (e.g., educational workshops, nutritional counseling), patients may lack critical knowledge about hypertension prevention, management, and the importance of lifestyle modifications. Meaningful and sustained communication between patients and providers about BP management, lifestyle changes, and medication adherence are ways to engage patients in care plans as they navigate community resources [64, 66]. Leveraging trusted community spaces (i.e., churches, barbershops, etc.) to implement therapeutic lifestyle change programs demonstrated methods to disseminate equitable care among the community [68, 69]. In addition, facilitating the development of inclusive, urban planning improves equitable distribution of resources to underserved neighborhoods [70].

### Policy Level

Implementation and dissemination of multiple policy-level modifications can improve health care outcomes, especially in the Black and minoritized populations. Despite the value of creating solutions at the health system and community levels, state and federal policies concurrently highlight the importance of accountability. Many federal and state initiatives have been implemented to support the health system and improve health outcomes. For example, limited or no insurance coverage decreases access to medications, BP monitoring devices, and visits while leading to higher out-of-pocket expenses. Evidence demonstrates that policies to reduce or eliminate patient out-of-pocket costs of antihypertensive medications improve hypertension control [44] and routine follow-up visits. Another solution to improve hypertension control is to create and implement policies that codify the equal distribution to or access to medical benefits for all employers and occupations [71]. Lack of policies to enhance provider incentives for BP control is another layer of reimbursement from the provider’s standpoint that impacts achievement of hypertension goals. Additionally, decreased funding to assist with health care supplemental coverage for co-pays for office visits or testing [72] also impacts hypertension control. Overall, policies aimed at expanding insurance coverage or improving health care quality successfully improved medication use and BP control among U.S. adults with hypertension [72]. Policies must acknowledge and stimulate awareness of structural racism as a fundamental cause of health care disparities while integrating cultural humility. Currently, there are a paucity of policies in place to eliminate inequities in access to quality health care. Evidence demonstrates systemic/structural racism leads to poorer health by increasing exposure to health-harming conditions and limiting access to health-promoting resources and opportunities [44]. Thus, creating and implementing policies to dismantle residential segregation and its effect on health outcomes while eliminating inequities in access to and quality of health care are necessary components to address disparities in outcomes [44].

Access to health education and standardized health information geared to the community is critical for scaffolding patient engagement and understanding. When there are barriers to accessing health education such as cost, location, or language, health disparities prevail and permeate health knowledge and behaviors across various populations. Policies must provide support for open health education with established guidelines or regulations ensuring that everyone can readily access quality health information and education. Black and minoritized populations face multiple barriers to education, deep-rooted in structural racism, and perpetuated by discriminatory policies that have historically marginalized the Black, Hispanic, and American Indian communities. The effect of poor education has led to poor health literacy and lack of access to relevant information on cardiovascular risk factors [73]. Policies aimed at developing interventions are needed to narrow the racial educational gap. These interventions should begin in childhood and must take a multi-faceted approach toward creating a more just and equitable education system [73]. Along with health education, minoritized communities also lack access to adequate nutritional content and are overexposed to unhealthy foods and ingredients. For instance, regulation on sodium content is lacking for food establishments and processed food companies [74]. Effective public policy on sodium reduction is central to improving hypertension and preventing CVD [74]. Income inequality is associated with elevated risk of hypertension [75], and income disparities are associated with unstable living conditions which compromise healthcare outcomes [39]. Policies that focus on establishing a livable wage to improve socioeconomic position among racially minoritized populations, are essential in reducing racial disparities in hypertension, and advancing cardiovascular health equity [75]. Moreover, providing accessible job training programs can empower individuals to achieve higher incomes to close the income disparity gap.

## Conclusions

The evidence of patient-physician communication on BP management demonstrates gaps in care particularly among the NHB patients. Although, healthcare in the U.S. boasts great innovations and successes in the past few decades, the color of a person’s skin determines—to a considerable extent—the quality of care received, his/her perception and forecast of health, disease and mortality risk. Racialized differences in hypertension result in NHB and other minoritized patients bearing a disproportionate burden of hypertension and CVD. The disadvantage experienced by Black individuals and other minoritized populations may be improved by bridging gaps in patient communication among clinicians, population health scientists, policymakers, and community members/leaders. To overcome this chasm, integration of evidence-based, multilevel efforts must be implemented at the patient, clinician, community, organizational and policy level to address the SDoH. Central to this process will be improved patient-clinician communication to promote optimal health for all. Future research and policy should prioritize scalable solutions to enhance patient-clinician communication and outcomes.

## Data Availability

No datasets were generated or analysed during the current study.
